# Evaluating the Potential of Gamma‐Glutamylcysteine and Glutathione as Substitutes for SO_2_ in White Wine

**DOI:** 10.1002/fsn3.70058

**Published:** 2025-02-17

**Authors:** Mumine Guruk, Patrick Fickers, Bilal Agirman, Merve Darıcı, Huseyin Erten

**Affiliations:** ^1^ Department of Food Engineering, Faculty of Engineering Cukurova University Adana Türkiye; ^2^ University of Liege—Gembloux Agro‐Bio Tech, TERRA Teaching and Research Centre Gembloux Belgium

**Keywords:** antioxidant, gamma‐glutamyl cysteine, glutathione, Narince, sulfur dioxide, white wine

## Abstract

Sulfur dioxide (SO_2_) is the most common additive used in winemaking with its antimicrobial and antioxidant properties. However, in recent years, there has been an increasing tendency to reduce the use of excessive SO_2_ in wine due to its negative effects on human health and organoleptic characteristics. Therefore, the aim of this study was to evaluate the potential use of glutathione (GSH) and its precursor, gamma‐glutamyl‐cysteine (gGC), which are known as strong antioxidants, for substituting SO_2_ in white wines. The Turkish indigenous white grape cultivar, Narince (*Vitis vinifera*), was used to produce white wine. The wines with additions (SO_2_, gGC, and GSH) and those without (control) were matured for 2 months. As a result, the protective effects of gGC and GSH additions on phenolic compounds, as well as their ability to reduce browning in white wine, were demonstrated. The addition of gGC and GSH enhanced the amount of esters and higher alcohols, improving the wine's aroma. In conclusion, gGC and GSH—particularly gGC—showed great potential as SO_2_ replacements in wine.

## Graphical abstract

Fermentation was carried out using the Narince (*Vitis vinifera*) grape variety. SO_2_ and gamma glutamyl cysteine and glutathione were added to the wine produced during bottling as a preservative for substituting SO_2_. There was no major difference in the proximate composition of wines supplemented with gGC, GSH, and SO_2_. Furthermore, it has been proven that the inclusion of gGC and GSH in wine proved to be efficacious in protecting phenolic compounds. It is worth noting that the addition of gGC slowed down the browning reactions and resulted in a browning degree value equivalent to SO_2_‐added wine. Furthermore, compared to the control and SO_2_ groups, the addition of gGC and GSH increases the amounts of esters and higher alcohols, which improves the aroma of the wine.

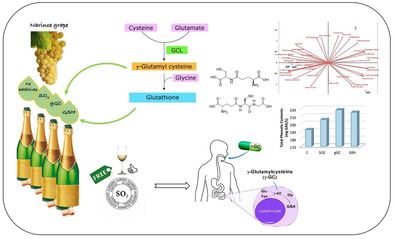

## 1 Introduction

Due to its antimicrobial and antioxidant properties, the colorless and pungent gas sulfur dioxide (SO_2_) is a widely used preservative in the wine industry (from must to wine production) (Giacosa et al. 2019). Moreover, it prevents both enzymatic and nonenzymatic processes (Oliveira et al. 2011). More SO_2_ is specifically utilized in the manufacture and storage of white wines to prevent oxidation reactions that alter the chemical composition and result in browning, which degrades the wine's sensory value.

Despite its antioxidant benefits, the European Union (EU) restricts the use of SO_2_ in wine due to health concerns. The maximum limits for total SO₂ in white and rosé wines are 200 mg/L and 150 mg/L for red wines, according to European Union regulations (EUR‐Lex, 2019). Furthermore, for organic wines, they are lower: white and rosé: maximum 150 mg/L total SO₂; red: maximum 100 mg/L total SO₂. In recent years, the presence of SO_2_ in wine has raised concerns about its adverse effects on health, such as individuals with asthma and chronic lung disease. As a result, enological research is seeking approaches to reduce or possibly replace SO_2_. The majority of studies on substitutes for SO_2_ have been focused on those with oxidative and antimicrobial characteristics (Lisanti et al. 2019; Bağder Elmacı et al. 2015).

Due to these factors, research has been conducted to determine whether SO_2_ levels can be decreased or whether additives such as tannin, lysozyme enzyme, and ascorbic acid can be utilized as alternatives in wine production for a long time (Lisanti et al. 2019). Recent research has focused on the antioxidant “glutathione” which has attracted interest due to its potential application as a SO_2_ substitute in wine. Two methods have been used to assess the glutathione effect in wine: adding it directly to the must or wine, or fermenting the wine using yeasts rich in glutathione (Kritzinger et al. 2013). However, research on glutathione supplementation is more prevalent.

γ‐Glutamyl cysteine (gGC, C_8_H_14_N_2_O_5_S) is a dipeptide comprised of cysteine and glutamate that forms in most living cells via glutamyl cysteine ligase (GCL) catalysis. Following the addition of glycine, glutathione synthetase converts it to glutathione (GSH) (Bachhawat and Yadav 2018). Studies on glutathione production by recombinant microorganisms have become more extensive as bioengineering has advanced, and studies on the recombinant production of glutamyl cysteine, its precursor, have gained interest in recent years.

The antioxidant activity of glutathione is attributed to the presence of cysteine and the ‐SH functional group amino acids that it contains (Elias et al. 2008). As glutamyl cysteine contains the amino acid cysteine, it is hypothesized that it will have an antioxidant effect similar to glutathione, despite the lack of research in this field in the literature.

The above‐mentioned reasons have led to the focus on glutamyl cysteine. Therefore, the present study aimed to evaluate the antioxidant capacity and potential of glutamyl cysteine and glutathione as possible alternatives for SO_2_ in white wine. Moreover, the effect of these two compounds on the physical, chemical, aroma, and sensory characteristics of 2‐month‐matured wine was investigated.

## 2 Materials and Methods

### 2.1 Reagents and Material

The grapes utilized in this study were of the Narince ( *Vitis vinifera* L., 1753) variety and were obtained from the Nevşehir region of Turkey. Narince is a unique and commercially important white wine grape that has a historical importance from the Hittite period. Narince grape produces straw yellow‐colored wines with floral and citrus aromas. Narince wine has good potential for aging due to its medium‐ to full‐bodied properties with good acidity character balanced by a moderate alcohol level (Arslan et al. 2018). The yeast, *Saccharomyces cerevisiae* SAUVY, used in the study was from Lallemend Co. (France). Glutathione (GSH), gamma‐glutamyl cysteine (gGC, Glu‐Cys), and all other chemicals were purchased from Sigma Aldrich Co. (St. Louis, MO, USA).

### 2.2 Winemaking, Preparation and Maturation of Wine Samples

The must obtained by pressing grape berries was stored at 10°C for 24 h. Then, the must was pre‐clarified by filtration using a filter press to obtain higher yields of clear juice. Before being added to the must, the Sauvy yeast was rehydrated according to the manufacturer's (Lallemand Oenology, France) specifications. Subsequently, *S. cerevisiae* SAUVY was dosed to the must at a concentration of 25 g/hL. The experiments were conducted in parallel sets with 3 L of must in 5 L glass drums. The fermentation was conducted at 18°C. The following parameters were used to monitor the fermentation over the course of the 12‐day process: total acidity using the NaOH standardization method (OIV 2022), brix using a hand refractometer (KEM Kyoto, RA‐130, Japan), density using a portable density meter (Mettler Toledo Densito 30PX, USA), and pH using a digital pH meter (Mettler Toledo S400, USA).

During alcoholic fermentation, total yeast (TY) and lactic acid bacteria (LAB) were enumerated on Days 0, 4, 8, and 12. Potato dextrose agar (PDA, Merck, Germany) supplemented with oxytetracycline (100 mg/L, Sigma‐Aldrich, USA) and De Man, Rogosa, and Sharpe agar (MRS, Merck, Germany) supplemented with cycloheximide (100 μg/mL, Sigma‐Aldrich, USA) were used to enumerate TY and LAB, respectively.

Following the fermentation process, the wine was divided into four groups. For the first three groups, 30 mg/L of gamma‐glutamylcysteine (gGC), glutathione (GSH), and SO_2_ were added to the wines separately. The fourth group, established as the control (C), proceeded with no additives. Prior to analysis, all wine samples had been matured in 300‐mL glass bottles at 10°C for 2 months. All experiments were set up in duplicate.

### 2.3 Chemical Analysis and Alcohol Determination

Density, dry matter, total acidity, and pH were monitored during the fermentation process. At the end of the 2‐month maturation period, volatile acidity, free SO_2_, and total SO_2_ were determined. All chemical analyses were conducted in accordance with the International Organization for Vine and Wine's International Methods of Wine and Must Analysis (OIV 2022).

Using an Anton Paar Alcolyzer (DMA 4500 M‐Alcolyzer ME, Austria) equipment, the amount of ethyl alcohol in wines was determined by means of NIR (near infrared) spectroscopy. The alcohol content is represented as a percentage (%) of the total volume (OIV 2022).

### 2.4 Determination of Total Phenolic Compounds

The total phenol content of wine samples was determined using a modified version of the Folin–Ciocalteu colorimetric method as described by Nardini and Garaguso (2018). Using a spectrophotometer (Perkin Elmer Lambda 25‐UV/VIS, USA), the absorbances were measured at 765 nm following a 30‐min dark incubation period. The calibration curve was prepared with a standard solution of gallic acid at several concentrations (0, 50, 100, 150, 250, and 500 mg/L). The results were expressed as mg gallic acid equivalents (GAE) per liter. All measurements were performed in triplicate.

### 2.5 Determination of Organic Acids and Reducing Sugars

Total and reducing sugars were determined by the Luff‐Schoorl method (OIV 2015). Organic acids (tartaric, malic, and citric) and glycerol were quantified by the HPLC (Shimadzu, LC‐20AT, Kyoto, Japan) system. Wine samples were filtered via a 0.45 μm PTFE filter (Sartorius, Germany) before injection. The system was composed of a quaternary pump, a column temperature control oven (CTO‐10AS), an auto sampler unit (SIL‐ 20A), a degasser module (DGU‐20A5), and a photodiode array detector (SPD‐M20A). The analysis was performed at a 210 nm wavelength using an Aminex HPX‐87H (300 × 7.8 mm, Bio‐Rad, USA) column.

Compounds were eluted using a 5 mM H_2_SO_4_ solution at a flow rate of 0.5 mL/min. HPLC analyses were performed as triplicates.

### 2.6 Determination of Browning and Measurement L*, a*, b*, Hue, Chroma by CIELAB

To determine the degree of enzymatic browning, a 10 mL sample was centrifuged at 4°C at 1000 rpm × g for 10 min. The supernatant (5 mL) was then combined with 5 mL of 95% ethyl alcohol and centrifuged under the same conditions. The absorbance of the resulting solution was measured at 420 nm against a blank (95% ethanol) in a spectrophotometer (Perkin Elmer Lambda 25‐UV/VIS, USA).

The color values (L*, a*, b*) of the wine samples were directly measured by the ColorQuest XE (3A/SB, USA) model HunterLab device. L* indicates the degree of light value and ranges from 0 (black) to 100 (white). The a* value indicates green (−a*) to red (+a*), and the b* value indicates blue (−b*) to yellow (+b*). The chroma and hue values are computed using the formulas described below.

Chroma = (a*^2^ + b*^2^)^1/2^ and Hue = tan^−1^ (b*/a*).

### 2.7 Sensory Evaluation of Wines

Sensory evaluation of wine samples was carried out after 2‐month storage. The rating test, as outlined by Meilgaard et al. (2007), was conducted by a panel of ten panelists comprising five males and five females, with ages ranging from 25 to 55. The Ethics Committee of Çukurova University's Food Engineering Department authorized permission and ethical approval for this study to conduct sensory panel research, and each panelist provided informed consent prior to the sensory test. The panelists were asked to score each attribute from low to high using a 9‐point hedonic scale (1: dislike extremely; 5: neither like nor dislike; 9: like extremely). The wine samples were judged based on their color (light yellow‐green, gold, and amber), odor (flowery, herbal tea, lavender, citrus, tropical fruits, tree fruits, honey, and herbaceous), and flavor (sourness, bitterness, sweetness, body, and harmony) criteria. During the sessions, International Standard Organization (ISO) wine glasses covered with glass petri dishes were used to serve the wine (20 mL at 20°C) in a random order.

### 2.8 Determination of Aroma Compounds by GC/FID/MS

Aroma compounds were determined with the Solid Phase Micro Extraction (SPME) technique by the “Agilent 7890B” GC equipped with a flame ionization detector (FID) and “Agilent 7010B” MS systems. Three mL of sample with 4‐nonanol (41.57 μg/5 μL as internal standard) in a 20 mL vial is maintained at 40°C for 10 min, and then aroma compounds were adsorbed for 30 min using a Solid Phase Micro Extraction (SPME) apparatus with a 50/30 μm Divinylbenzene/Carboxene/Polydimethylsiloxane (DVB/CAR/PDMS, 2 cm) coated fiber. The fiber was then injected into the DB‐Wax (60 m × 0.25 mm i.d. × 0.25 m, J&W Scientific‐Folsom, USA) capillary column following desorbing for 5 min. The injection temperature was set to 250°C. The column temperature was increased by 2°C per minute to 90°C after 4 min of holding at 40°C, then to 130°C by increasing 3°C per minute, and finally to 240°C by increasing 4°C per minute and holding at this temperature for 15 min. Helium was used as the carrier gas at a flow rate of 1 mL/min. The electron energy was 70 eV, the mass range was 30–600 m/z, the scan rate was 1.0 scans, the interface temperature was 250°C, and the source temperature was 120°C. The split ratio is 1:10. The aroma compounds were identified by comparing their retention index and mass spectra on the DB‐Wax column with those of a commercial spectra database (W10N14, NIST11, NBS 75k) and the instrument's internal library, which was compiled from prior experimental studies (Darıcı and Cabaroglu 2022; Agirman et al. 2024). Using an n‐alkane series (C8–C26), retention indices of the compounds were computed. Following the identification of aroma compounds, the internal standard procedure was used to quantify the aroma compounds (Schneider et al. 2004; Eker et al. 2023). The ratio of peak area was corrected using response factors for each compound, which were determined using the intensity ratio of each compound to the internal standard.

### 2.9 Statistical Analysis

The data obtained from the analyses were evaluated using a one‐way analysis of variance (ANOVA) with the use of IBM SPSS Statistics software, version 23.0, developed by IBM Corporation in Armonk, NY, USA. Duncan's multiple‐range tests were applied to assess the statistically significant differences between the mean values, with a significance level of *p* < 0.05. XLSTAT (2023) software was used to create a biplot graph for principal component analysis (PCA).

## 3 Results and Discussion

### 3.1 Monitoring Alcohol Fermentation and Determination of Microbial Population

**FIGURE 1:**
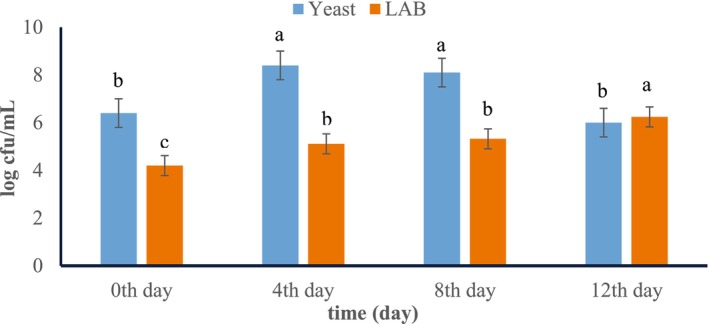
Total yeast and LAB growth during alcohol fermentation with must without SO_2_. The first column indicates total yeast and the second column total LAB (*n* = 2, *p* < 0.05). Different letters within the columns of the same microbial group represent statistically significant differences (*p* < 0.05) among the different times of fermentation.

The growth of yeast and LAB was followed during fermentation. However, LAB and molds can not grow in the presence of SO_2_; depending on the concentration, yeasts are not generally affected. LAB in the wine is desirable to conduct malolactic fermentation. LAB are also sensitive to SO_2_. Enumeration of yeasts and LAB during SO2‐free alcohol fermentation is shown in Figure 1.

*Saccharomyces cerevisiae* is the key microorganism in wine due to its satisfactory fermentative capacity, high ethanol productivity, rapid growth, easy adaptation, high tolerance to SO_2_, and production of numerous sensorially active compounds (i.e., esters, higher alcohols, volatile acids, etc.) (Maicas 2020). Fermentation started with 6.4 log cfu/mL. While the number of yeast increased until the 8th day of fermentation, it then started to decrease and reached 6 log cfu/mL at the end of fermentation (*p* < 0.05). Yeast growth demonstrated the same trend as the study of Çelik et al. (2019), which produced white wine from Narince grapes using autochthonous and commercial *S. cerevisiae* strains.

High SO_2_ supplementation (> 20 mg/mL) has been shown to decrease LAB growth (Capozzi et al. 2021). However, in this investigation, in which SO_2_ was not employed, the LAB population was 4.2 log cfu/mL at the start of fermentation and progressively rose over the course of the following days (*p* < 0.05). At the end of the fermentation, it reached 6.24 log cfu/mL. In other studies, the population of LAB generally starts at 3 log cfu/mL on the first day of fermentation and then increases, as in this study (Lafon‐Lafourcade et al. 1983; Petri et al. 2013; Hasalliu et al. 2017). Subsequently, the initiation of malolactic fermentation occurs at the level of a population of LAB reaching 6 log cfu/mL (Lonvaud‐Funel 1999). Although a slightly higher LAB number was determined in SO_2_‐free wine in this study, these findings were consistent with the previous studies reported above.

### 3.2 Composition of SO_2_ ‐Free Wine

The quality of a wine is determined by its chemical composition, which includes parameters such as ethanol content, residual sugars, total and volatile acidity, organic acids, color, phenolic compounds, and aroma compounds. Table 1 describes the physico‐chemical composition of the must and wines that were produced.

**TABLE 1:** Compositon of Narince grape must and white wine.

*Composition of must*	
Density (g/cm^3^)	1.125 ± 0.02
Brix (%)	24.8 ± 0.00
pH	3.03 ± 0.05
Total acidity (g/L)	5.66 ± 0.05
Total sugar (g/L)	230.5 ± 0.02
*Composition of wine*	
Density (g/cm^3^), 20°C	1.002 ± 0.001
Alcohol (%volume), 20°C	12.50 ± 0.03
Total acidity (g/L)^1^	6.73 ± 0.17
pH	3.03 ± 0.06

Composition of wine samples after storage					
	Control	SO_2_	gGC	GSH	
Glycerol (g/L)	7.12 ± 0.19^a^	6.62 ± 0.14^b^	6.85 ± 0.15^a,b^	6.80 ± 0.08^b^	*
Reducing sugar (g/L)	3.62 ± 0.00	3.62 ± 0.00	3.62 ± 0.03	3.62 ± 0.00	ns
Total Sugar (g/L)	3.86 ± 0.00^a^	3.77 ± 0.00^c^	3.79 ± 0.00^b^	3.80 ± 0.05^b^	*
Tartaric acid (g/L)	3.32 ± 0.08^b^	3.41 ± 0.12^a,b^	3.40 ± 0.06^b^	3.57 ± 0.00^a^	*
Malic acid (g/L)	2.18 ± 0.06^b^	2.45 ± 0.03^a^	2.20 ± 0.00^b^	2.20 ± 0.00^b^	***
Citric acid (g/L)	0.61 ± 0.1^b^	0.63 ± 0.02^a^	0.60 ± 0.01^b,c^	0.60 ± 0.06^c^	**
Volatile acidity (g/L)^2^	0.60 ± 0.00^a^	0.55 ± 0.00^b,c^	0.52 ± 0.01^c^	0.56 ± 0.01^b^	***
Free SO_2_ (mg/L)	6.40 ± 0.5^c^	19.20 ± 0.5^a^	13.33 ± 0.5^b^	13.86 ± 0.5^b^	***
Total SO_2_ (mg/L)	12.80 ± 1.6^d^	57.06 ± 1.6^a^	33.06 ± 1.6^c^	40 ± 1.6^b^	***
*Color*					
L*	88.45 ± 0.15^d^	89.32 ± 0.08^c^	90.39 ± 0.29^b^	90.80 ± 0.08^a^	***
a*	−0.46 ± 0.01^d^	−0.14 ± 0.01^a^	−0.22 ± 0.01^c^	−0.20 ± 0.00^b^	***
b*	9.72 ± 0.08^c^	11.54 ± 0.06^a^	11.39 ± 0.08^b^	11.47 ± 0.02^a,b^	***
Chroma (C)	9.73 ± 0.08^c^	11.54 ± 0.06^a^	11.39 ± 0.08^b^	11.47 ± 0.02^a,b^	***
Hue (°)	92.71 ± 0.12^a^	90.70 ± 0.04^c^	91.14 ± 0.08^b^	90.00 ± 0.00^b^	***

*Note:* Data shown with different letters (a, b, c) within lines are significantly different by Duncan (*p* < 0.05). 1 = as tartaric acid, 2 = as acetic acid.

Abbreviations: C, control; gGC, gamma glutamyl cysteine and GSH: glutathione; SO_2_, sulfur dioxide.

* *p* < 0.05.

** *p* < 0.01.

*** *p* < 0.001.

Glucose and fructose, which are referred to as reducing sugars, constitute the primary sugars found in grapes. The reducing sugar content in grape must is between 150 and 250 g/L (Alnuwaiser 2017). Sugars, being fundamental nutrients for yeasts during the process of fermentation, are a crucial factor that influences the final form of the product (Jakabová et al. 2021). The Narince grape, which is widely recognized as a prominent white wine grape variety in Turkey, is predominantly grown in the Nevşehir and Tokat districts. The current study involved conducting fermentation using Narince grape‐must characterized by a dry matter content of 24.8%, total acidity of 5.66 g/L tartaric acid, pH of 3.03, and sugar content of 230.5 g/L.

The reducing sugar level in wine samples was determined to be 3.62 g/L. There was no statistically significant difference observed in the sugar content of the wines among the C, SO_2_, GC, and GSH groups. According to the EU regulation 753/2002, wines are classified as dry (< 4 g/L), medium dry (< 12 g/L), medium (< 45 g/L), and sweet (> 45 g/L) based on sugar content. The total sugar content of the wines was C, SO_2_, GC, GSH; 3.86, 3.77, 3.79, 3.80, respectively.

The alcohol content of all samples was determined as 12.5% by volume. The alcohol content of wine produced from the Narince grape often ranges within the range of 10%–12% (Arslan et al. 2018; Çelik et al. 2019).

The component glycerol, which is produced by yeasts during the fermentation of alcohol, contributes to the sweetness, smoothness, and complexity of wine (Gao et al. 2019). Additionally, glycerol plays a significant role in enhancing the feeling of fullness of the body in wine (Zhao et al. 2015). The quantity of glycerol is dependent on the sugar concentration present in the must as well as the temperature at which fermentation occurs. The glycerol yield can be improved by elevated levels of sugar content and fermentation temperature (Ivit et al. 2020). In this context, the amount of glycerol was consistent with the initial sugar level, as seen in Table 1. It was determined that the corresponding glycerol concentrations for C, SO_2_, GC, and GSH were 7.12 g/L, 6.62 g/L, 6.85 g/L, and 6.80 g/L, respectively. The control sample exhibited a statistically significant difference when compared to the wine with additives (*p* < 0.05).

Typically, the concentration of glycerol in white wines is approximately 7 g/L (Ivit et al. 2020). The glycerol contents of white wines produced from must with 20.4 brix ranged from 5.93 to 7.37 g/L in a study by Arslan et al. (2018) that used the same grape variety (Narince) and fermentation temperature (18°C) as our investigation. In another comparative study (García et al. 2020), glycerol content varied from 7.06 to 9.30 g/L in white wines produced using the Malvar grape variety, fermented at 20°C, and including 230 g/L reducing sugar in must. As a result, the recorded amount of glycerol in the current investigation was consistent with previously published studies on white wines.

Organic acids, which are abundant in fermented foods, are compounds that contribute to food quality and organoleptic properties. Organic acids in wine serve to impart physicochemical and microbiological stability (Robles et al. 2019). The aroma of wine is also influenced by organic acids (Bae et al. 2014). The main organic acids found in wine are tartaric, malic, citric, succinic, and acetic acids (Nascimento Silva et al. 2015).

Tartaric acid is a critical factor in determining the overall acidity of wine and plays a significant role in preserving its chemical stability, color, and flavor. The concentration of tartaric acid may decrease due to the occurrence of precipitation in the form of tartaric crystals during the process of fermentation (Ivanova‐Petropulos et al. 2018). The amounts of tartaric and malic acid in the control, SO_2_, gGC, and GSH wine samples were 3.32, 3.41, 3.40, 3.57 and 2.18 g/L, 2.45, 2.20, and 2.20 g/L, respectively. Among the volatile acids, citric acid records the lowest concentration. Between wine samples, there was no significant difference between the gGC and GSH samples. The highest concentration was determined in (0.63 ± 0.02) SO_2_ sample. Volatile acids are chemical compounds that are produced through the process of alcohol fermentation, with acetic acid being a particularly significant example. When the wine samples were examined, the gGC sample had the lowest volatile acid in terms of acetic acid, with 0.52 g/L, and the control sample had the highest (0.6 g/L). The difference between the volatile acid values was significant (*p* < 0.05) (Table 1).

SO_2_ present in wine can be categorized into two distinct groups: bound and free forms. Bound SO_2_ contains aldehydes, ketones, and phenolic derivatives, while free SO_2_ is H_2_SO_3_, HSO_3_,^−^ and SO_3_^2−^. The International Organization of Vine and Wine (OIV) has been gradually decreasing the permissible total SO_2_ in wines due to the health issues linked to its usage (Santos et al. 2012). Wines with a high sugar content have a higher SO_2_ limit because they deteriorate quicker. The established threshold for demi‐sec wines is 275 mg/L. The free and total sulfur content of the wines was determined to be 19.20; 57.06, 13.86; 40.00, 13.33; 33.06, 6.40; 12.80 mg/L in the following order: SO_2_, GSH, GC, C (*p* < 0.05). The quantity of SO_2_ is a subject of significant concern. A study was conducted to evaluate the SO_2_ content of 316 wine samples. The recorded values, ranging up to a maximum of 340 mg/L, were then compared based on the countries of origin and the varietals of wine. German (113.85 mg/kg) and Italian (113.58 mg/kg) wines had lower SO_2_ levels than Chinese (193.17 mg/kg) and Korean (340 mg/kg) wines, whereas white (122 mg/kg) wines have higher SO_2_ levels than red (71.13 mg/kg) wines. The World International Organization of Vine and Wine (OIV) limited SO_2_ in wine to 300 mg/L (> 4 g/L containing reducing substances), 400 mg/L (certain sweet white wines) (Kontaxakis et al. 2020).

### 3.3 Determination of Total Phenolic Compounds in Wine Samples

Phenolics are the most important secondary metabolites found in plants, influencing wine quality. Phenolics are divided into two groups: flavonoids and non‐flavonoids. The compounds are present in grape skin, seeds, and juice. These compounds vary by grape type, geographical location, fermentation conditions, and post‐fermentation processes. As phenolic compounds have an effect on the color and organoleptic properties of wine, they should be preserved until consumption. Total phenol content was determined to be 192.23, 205.57, 215.22, and 218.43 GAE/L in mature wine for C, SO_2_, GSH, and gGC wines, respectively. While there is no statistically significant difference between gGC and GSH, it is worth noting that these two groups have a higher value than wine containing SO_2_.

The total phenolic levels were comparable to those reported by Vaimakis and Roussis (1996) who reported total phenolic values in white wine with added glutathione ranging from 110 to 150 mg GAE/L. In the present study, GSH and gGC wine samples had higher phenol content. However, according to Bayram and Kayalar (2018), who worked with Narince grapes harvested from two different localities (Emirseyit and Erbaa), the total phenolic content (443 and 403 GAE/L for Erbaa and Emirseyit wines) was higher than that in this study. According to the findings of Jakabová et al. (2021), the concentrations of total phenolic substances in conventional and SO_2_‐free wines were 273 and 285 mg GAE/L, respectively. In the white wine with the addition of glutathione, total phenol content was measured in the range of 241–449 mg caffeic acid by El‐Hosry et al. (2009). In addition to preventing phenolic substance loss, glutamyl cysteine and glutathione consumption are important for consumer health.

### 3.4 Browning and Color Determinations in White Wines

**FIGURE 2:**
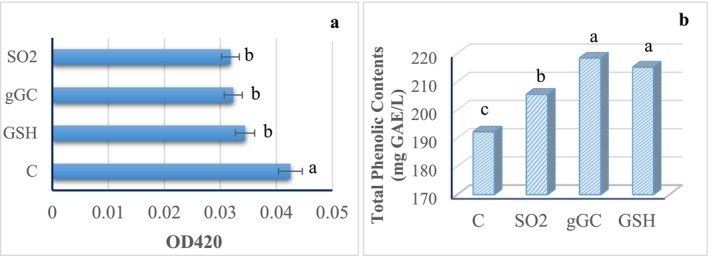
The influence of SO_2_, gGC, and GSH additions to white wine during maturation on browning degree (a) and total phenolic content (b). C (control, without additives), SO_2_ (30 mg/L), gGC (gamma glutamyl cysteine, 30 mg/L), GSH (glutathione 30 mg/L). Data shown with different letters (a, b, c) within lines are significantly different by Duncan (p < 0.05).

Browning consists of enzymatic and non‐enzymatic reactions. A green‐brown color is observed as a result of the oxidation of phenolic substances in wine. This is particularly undesirable in young wines. Browning is usually determined by measuring the color at 420 nm. This parameter is related to the decrease in SO_2_ concentration in wine. The OD_420_ values obtained in this investigation indicated that SO_2_ (0.031 ± 0.004) and gGC (0.032 ± 0.001) exhibited the lowest values. A low OD_420_ value indicates a reduced degree of browning. The OD_420_ absorbance values of GSH and control samples were determined to be 0.034 ± 0.001 and 0.042 ± 0.002, respectively (Figure 2). SO_2_ and gGC were identified as the most efficacious additives in minimizing browning, as indicated by the results. All three groups differed significantly from the control group (*p* < 0.05). It is worth noting that gGC has a greater browning‐reducing impact than GSH. While no reported data exist regarding the impact of gGC on browning in wines, the SO_2_ and GSH values were consistent with those reported in the literature (Motta et al. 2022). According to El‐Hosry et al. (2009) wine and the addition of GSH resulted in a considerable decrease in browning degree even on Day 0. Consequently, it has been demonstrated that GSH significantly affects the stability of wine when exposed to oxidation. Although the components responsible for browning have not been identified in white wines, diphenols that cause browning have not been identified; diphenol compounds are known to be the most susceptible to browning (Giménez et al. 2022). Consequently, protecting phenolic substances is critical for maintaining color stability (Milat et al. 2019).

Table 1 demonstrates changes in the color of the wines as a measure of the color (L*: brightness/darkness, a*: greenish/redness and b*: blueness/yellowness) of the samples. In the experiments, the L* values were ranked from highest to lowest as GSH, gGC, SO₂, and C, respectively. The L*, a*, and b* values were determined to be 90.80, −0.20, and 11.47 in the GSH‐added sample; 90.39, −0.22, and 11.39 in the gGC‐added sample; 89.32, −0.14, and 11.54 in the SO_2_‐added sample; and 88.45, −0.46, and 9.72 in the control sample.

When the results of all the experiments are compared, the L* value is lower, and the b* value is higher than the values reported in the literature (Recamales et al. 2006). The color results in this study indicated that all produced wines, including the control group, had a clear pale yellow color, which is comparable to the study of Cosme et al. (2019), who reported that the color of the white wines changed from a clear pale yellow to light salmon.

**FIGURE 3:**
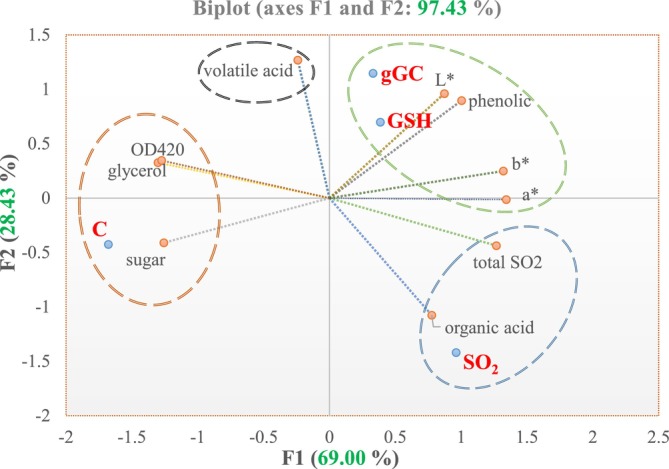
Bi‐plot of PCA based on the chemical composition of Narince wine samples (C: control = without additives, SO_2_: 30 mg/L, gGC: gamma glutamyl cysteine = 30 mg/L, GSH: glutathione = 30 mg/L).

The application of principal component analysis (PCA) was employed to provide a more comprehensive elucidation of the chemical composition of Narince wines based on different additions. The total variance explained by two principal components was 97.43%, with the first component (F1) comprising 69.00% and the second component (F2) 28.43% (Figure 3). gGC and GSH treatments were separated along PC2 (upper right quadrant) and displayed a positive correlation with each other. These two treatments were characterized by high L* and total phenolic compounds values. This indicates that GSH and gGC were successful in preserving the level of phenolic substances. The wine containing SO_2_ is located in the bottom right quadrant and is explained by its high organic acid (tartaric, malic, and citric acid) contents, consistent with the results given in Table 1. As can be seen from Figure 3, the control wine sample was located in the bottom left quadrant and characterized by high sugar and glycerol content.

### 3.5 Determination of Aroma Compounds by SPME GC/MS

**FIGURE 4:**
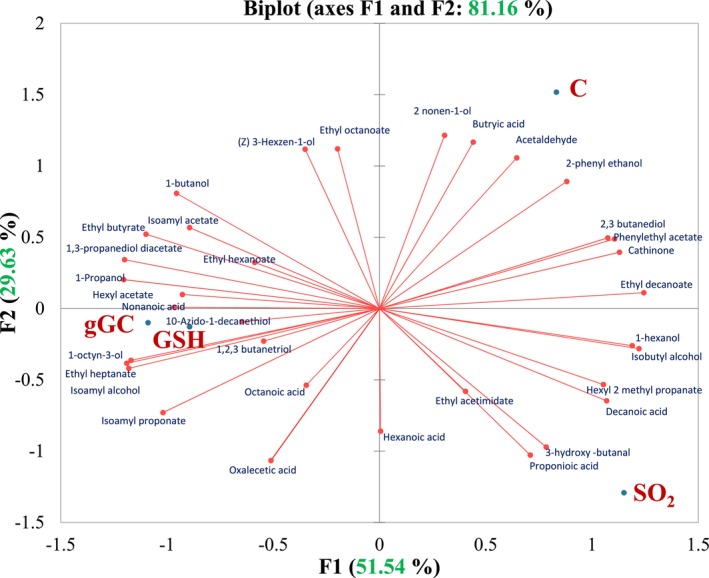
Principal component analysis of quantified volatile compounds of four wine samples: C (control, without additives), SO_2_ (30 mg/L), gGC (gamma glutamyl cysteine, 30 mg/L), GSH (glutathione 30 mg/L).

The volatile components of SO_2_, gGC, GSH added, and C wines are shown in Table 2. A total of 34 volatile components were determined by the SPME method. Table 2 lists the total volatile organic compounds (VOC) that were detected: eleven higher alcohols, twelve esters, seven volatile acids, three carbonyl compounds, one alkane, and one thiol. This is also illustrated in the PCA biplot in Figure 4.

**TABLE 2:** Volatile composition of wine from Narince grape with different additives.

Volatile compounds (μg/L)						
Higher alcohols	RI	C	SO_2_	gGC	GSH	*F*
1‐Propanol	1037	1082.92 ± 3.3^c^	1026.76 ± 2.68^d^	1193.93 ± 4.22^a^	1157.55 ± 6.32^b^	***
Isobutyl alcohol	1085	9824.06 ± 15.89^b^	10,626.05 ± 19.44^a^	8988.51 ± 10.9^c^	8975.82 ± 0.87^c^	***
1‐butanol	1165	606.18 ± 0.08^b^	521.54 ± 8.87^c^	636.17 ± 11.09^a^	618.99 ± 7.84^a,b^	***
Isoamyl alcohol	1210	102,109.28 ± 21.2^c^	112,865.66 ± 612.12^b^	124,664.86 ± 277.2^a^	125,795.3 ± 462.4^a^	***
1‐hexanol	1370	1786.61 ± 0.55^b^	1961.89 ± 6.94^a^	1498.26 ± 5.57^c^	1550.16 ± 8.71^c^	***
(Z)3‐Hexzen‐1‐ol	1401	118.36 ± 0.19^a^	103.28 ± 2.94^c^	116.02 ± 0.73^a^	109.5 ± 1.76^b^	***
2,3 butanediol	1495	1016.91 ± 2.14^a^	965.37 ± 0.09^b^	852.56 ± 0.08^d^	918.79 ± 1.82^c^	***
1,2,3 butanetriol	2062	295.81 ± 0.66^c^	321.01 ± 4.96^b^	290.76 ± 2.45^c^	467.66 ± 2.33^a^	***
1‐octyn‐3‐ol	1719	1850.65 ± 0.6^c^	2205.66 ± 6.59^b^	3162.22 ± 16.99^a^	3315.32 ± 18.1^a^	**
2‐phenyl ethanol	1916	29,526 ± 390.3^a^	29,323.97 ± 88.34^a^	25,889.43 ± 3.48^b^	25,910.2 ± 3.01^b^	**
2 nonen‐1‐ol	1804	1673.69 ± 4.65^a^	1329.61 ± 43.68^c^	1491.04 ± 1.47^b^	1520.51 ± 0.41^b^	***
Sum		149,890.47	161,250.8	168,783.76	170,339.8	
Esters						
Isoamyl acetate	1119	1951.46 ± 10.5^b^	1830.56 ± 9.67^c^	1946.76 ± 15.3^b^	2054.36 ± 49.5^a^	**
Ethyl hexanoate	1241	1185.95 ± 7.66^b^	1109.55 ± 2.98^c^	1134.55 ± 5.22^b,c^	1324.95 ± 44.34^a^	**
Isoamyl propionate	960	700.4 ± 0.01^b^	1342.46 ± 0.27^a^	1340.24 ± 0.08^a^	1332.56 ± 0.08^a^	**
Hexyl acetate	1250	266.7 ± 2.09^b^	202.1 ± 0.01^c^	596.7 ± 1.91^a^	286.7 ± 8.99^a^	**
Hexyl 2 methyl propanate	2891	1870.12 ± 0.37^b^	2142.56 ± 0.01^a^	1612.7 ± 0.33^d^	1824.64 ± 0.03^c^	***
Ethyl octanoate	1430	1164.4 ± 12.13^b^	369.25 ± 0.38^c^	816.8 ± 0.78^c^	1383.65 ± 16.5^a^	**
Ethyl decanoate	1635	336.5 ± 0.95^b^	342.62 ± 4.57^a^	123.75 ± 2.63^c^	167.12 ± 0.15^c^	***
Ethyl butyrate	1037	813.02 ± 0.01^b^	716.46 ± 0.08^c^	902.2 ± 0.46^a^	844.34 ± 0.04^a^	***
1,3‐propanediol diacetate	1189	153.68 ± 0.79^b^	126.65 ± 7.71^c^	192.27 ± 8.58^a^	180.33 ± 8.34^a^	**
Phenylethyl acetate	1785	712.25 ± 0.06^a^	615.5 ± 0.51^b^	401.25 ± 1.15^d^	506.5 ± 1.62^c^	***
Ethyl acetimidate	1091	210.2 ± 0.03^c^	300.56 ± 0.17^b^	348.43 ± 0.25^a^	307.5 ± 0.02^b^	**
Ethyl heptanate	1511	122.8 ± 0.06^c^	170.24 ± 0.04^b^	306.54 ± 0.42^a^	292.36 ± 0.0^a^	**
Sum		9487.48	8976.51	9722.19	10,485.01	
Volatile acids						
Proponioic acid	1538	33.93 ± 0.75^b^	44.71 ± 2.70^a^	34.59 ± 0.4^b^	34.08 ± 2.15^b^	*
Decanoic acid	2183	372.4 ± 0.01^b^	876.62 ± 1.02^a^	169.24 ± 0.05^c^	153.44 ± 0.29^c^	**
Nonanoic acid	2158	212.5 ± 0.04^c^	141.56 ± 0.06^d^	365.52 ± 0.95^b^	762.84 ± 0.01^a^	***
Butryic acid	1628	573.24 ± 0.13^a^	342.86 ± 0.28^c^	361.2 ± 0.01^b,c^	389.02 ± 0.1^b^	**
Octanoic acid	2060	1163.3 ± 0.25^c,d^	1452.04 ± 1.23^b^	1191.44 ± 1.35^c^	1833.23 ± 0.43^a^	***
Hexanoic acid	1840	1790.2 ± 0.01^b^	3345.06 ± 0.49^a^	3350.45 ± 0.33^a^	1800.4 ± 0.0^b^	**
Oxalecetic acid	2263	0.89 ± 0.04^c^	1.48 ± 0.35^b^	1.54 ± 0.0^a^	1.57 ± 0.41^a^	**
Sum		4145.16	6204.33	5473.78	4974.58	
Carbonyl compounds						
Acetaldehyde	500	5050.24 ± 0.15^a^	4700.5 ± 0.0^b,c^	4650.04 ± 0.01^c^	4750.22 ± 0.02^b^	**
Cathinone	1749	1155.3 ± 0.09^a^	1070.68 ± 1.19^b^	930.66 ± 0.17^c^	935.54 ± 0.01^c^	ns
3‐Hydroxy butanal	1325	150.45 ± 0.01^b^	192.08 ± 0.01^a^	153.44 ± 0.02^b^	151.24 ± 0.06^b^	*
Sum		6355.9	5963.26	5734.14	5837	
Thiol derivative						
10‐Azido‐1‐decanethiol	1671	nd	nd	nd	1.47	

*Note:* C (Control, without additives), SO_2_ (30 mg/L), gGC (gamma glutamyl cysteine, 30 mg/L), GSH (glutathione 30 mg/L). Data shown with different letters (a, b, c) within lines are significantly different by Duncan (p < 0.05).

Abbreviations: nd, not detected; ns, not significant.

* *p* < 0.05.

** *p* < 0.01.

*** *p* < 0.001.

Higher alcohols were the most abundant compounds in total and contributed to the aroma along with other flavor‐active compounds. They can also promote ester production. The samples exhibited significant variations in the total amount of higher alcohols (*p* < 0.05). The cumulative concentrations of higher alcohols in the C, SO_2_, gGC, and GSH experiments were 149.890, 161.250, 168.783, and 170.339 mg/L, respectively. The two most abundant compounds in total VOCs in all samples were 2‐phenyl ethanol and isoamyl alcohol. The presence of higher alcohols has a positive effect on wine up to 300 mg/L, while over 400 mg/mL has a detrimental effect (Swiegers et al. 2005). One of the most common higher alcohols in wine, 2‐phenyl ethanol, contributes to the wine's aroma with a sweet and flowery character. In this study, the highest concentration of phenyl ethanol was found in the control wine, followed by SO_2_‐added wine. The main higher alcohol found in this study was isoamyl alcohol (2‐methyl‐1‐butanol). It was found in high and close concentrations in both GSH (125.795 mg/L) and gGC (124.664 mg/L)‐added wines. Butanediol gives a buttery and creamy aroma that varies by wine type and has a direct effect on wine aroma. The amount of 2,3‐butanediol showed a higher concentration in C (1.016 mg/L) and SO_2_ (0.965 mg/L) in comparison to the GSH (0.918 mg/L) and GC (0.852 mg/L) added wines. The amount of 1‐hexanol also showed significant differences among the four samples (*p* < 0.05). 1‐hexanol affects wine with a resiny and flowery aroma.

Esters are aroma compounds that are present in the highest amounts in wine VOCs and give a characteristic fruity taste to the wine. The most common esters in wine are acetates. Isoamyl acetate, also known as 1‐butanol‐3‐methyl acetate, is an ester formed from acetic acid and isoamyl alcohol. It is known for having a strong banana flavor. Compared to the SO_2_ group, the GSH and gGC samples have greater levels of isoamyl acetate. The results for all samples had lower concentrations, as stated in the (Çelik et al. 2019; Swiegers et al. 2005). 3‐methyl‐propanoate (isoamyl propionate) is a carboxylic ester that has a sweet and bitter taste. The comparison test revealed that there was a difference with treatment C regarding the amount of isoamyl propionate, but not between treatments SO_2_, GSH, or gGC. Ethyl butyrate, ethyl hexanoate, ethyl decanoate, and ethyl octanoate are derived from the condensation reaction between organic acids and alcohol. They enhance the wine's fragrance by imparting a fruity flavor (Scutarașu et al. 2022).

Esters are produced by yeast during fermentation and contribute to the characteristic flavor of wines. The large group (12 individual compounds) of volatile organic compounds (VOCs) in wine samples consisted of quantified ethyl esters (ethyl butyrate, ethyl decanoate, ethyl hexanoate, ethyl heptanate, ethyl acetimidate and ethyl octanoate) and acetates (isoamyl acetate, isoamyl propanate, hexyl acetate, hexyl 2‐metil propanate, 1–3 propanediol diacetate and phenylethyl acetate). Isomethyl acetate and phenylethyl acetate contribute to the fruit jam aroma in wine (Antonelli et al. 1999). The threshold values of isoamyl acetate (3‐methyl butyl acetate), ethyl hexanoate, and ethyl octanoate are 30 μg/L, 50 μg/L, and 20 μg/L, respectively (Swiegers et al. 2005). According to the results obtained, these compounds are above the threshold value. However, the results in this study were lower than those in Bayram and Kayalar (2018).

According to the total ester content, the highest group was GSH (10.485 mg/L), followed by gGC (9.722 mg/L), C (9.487 mg/L), and the lowest was SO_2_ (8.976 mg/L).

Volatile acids are related to negative characteristics in wine, such as rancid and fatty, but they are also important for the aromatic equilibrium of wine (Belda et al. 2017). All samples included a low amount of total volatile acid concentration, as in other studies such as Selli et al. (2006). It was noteworthy that C wine (4.145 mg/L) contained a smaller amount of volatile acids than the others.

1‐alkyl thiols, sweets, and tropical factors decreased from a peak at 1‐heptane thiol. Up to 2 mg/L of decan compound has a tropical aroma feature; over 4 mg/L, it can produce unwanted fishy aromas (Sakoda and Hayashi 2002). 1‐Decanethiol was only detected in wine supplemented with GSH at a concentration of 1.47 μg/L.

Figure 4 displays the correlation biplot illustrating the relationship between volatile chemicals and the wine samples. All 34 VOCs were analyzed using PCA to determine how the samples differed from one another in terms of volatile content. With a total variance of 81.16%, the first two principal components, PC1 (51.54%) and PC2 (29.63%), were expressed.

**FIGURE 5:**
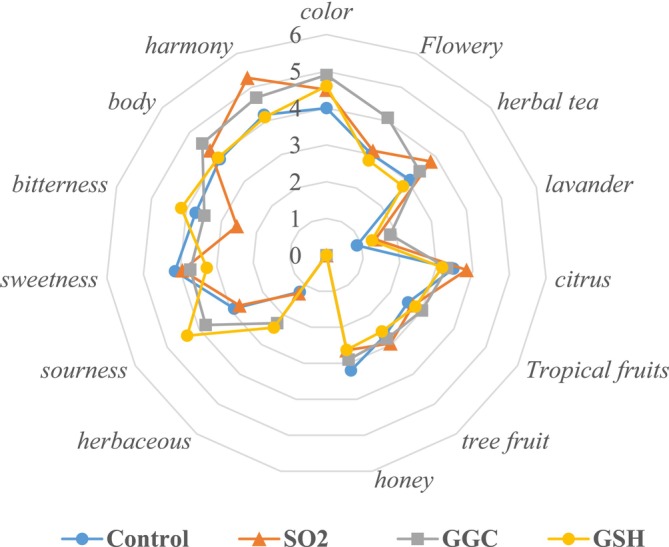
Sensory profile of wine samples. C (control, without additives), SO_2_ (30 mg/L), gGC (gamma glutamyl cysteine, 30 mg/L), GSH (glutathione 30 mg/L). No statistically significant difference was found by Duncan at the *p* < 0.05, *p* < 0.05, *p* < 0.01, and *p* < 0.001 levels between treatments for each attribute used in sensory analysis.

The total variance explained by two principal components was 81.16%, with the first component (F1) comprising 51.54% and the second component (F2) 29.63% (Figure 5). As can be seen from Figure 4, four samples were distributed among three distinct quadrants. However, gGC and GSH were included in the same quadrant (bottom left). Control wine was separated in the upper right quadrant and associated mainly with 2‐phenyl ethanol and 2‐nonen‐1‐ol, and 2,3‐butanediol as higher alcohols; acetaldehyde and cathinone as carbonyl compounds; phenylethyl acetate and ethyl decanoate as esters; and butyric acid as a volatile acid group. The wines with GSH and gGC were located close to each other (left bottom quadrant) in the bi‐plot graph, and they were correlated to the esters and thiol compounds. The SO_2_ group wines separated in the bottom right quadrant and explained mainly by volatile acids such as propionic acid, decanoic acid, and hexanoic acids. There was a positive correlation between higher alcohols and organic acids (r = 0.80, data not shown), and alkanes and higher alcohols (r = 0.98, data not shown). According to the VOC groups, higher alcohols, organic acids, and alkanes were close to each other while staying far from esters and thiols.

### 3.6 Sensory Analysis of Wine

The odor and flavor profiles of wine samples reported on the spider web diagram are shown in Figure 5.

On a 9‐point scale, the color value indicates the colors yellow‐green, gold, and amber from low to high. As a result, all wine samples were in the yellow‐gold range. The corresponding color scores for C, SO_2_, gGC, and GSH were 4.00, 4.50, 4.90, and 4.60, respectively.

In an evaluation of the wines based on their odor profiles, tropical fruit, flowery, and citrus characteristics predominated. SO_2_ obtained the highest value in the citrus profile (3.83), whereas gGC had the highest values for flowery and tropical fragrances (4.09 and 3.00, respectively).

The last criterium was about the evaluation of taste. The judges determined that GSH possessed the highest values for sourness and bitterness, scoring 4.37 and 4.15, respectively. In contrast, sample C exhibited the highest sweetness with a score of 4.14. In the assessment of the wines' body properties, the control sample exhibited the lowest value (3.9) compared to the gGC‐added wine, which exhibited the maximum value (4.54). The wines were generally ranked in the following order of appreciation: SO_2_, gGC, GSH, and C.

Although the addition of gGC has not been tried in wine before, the citrus, exotic fruit, honey, and caramel properties of GSH‐added wine (Panero et al. 2015) showed statistically significant differences among themselves, while a decrease in citrus flavor was observed in wine.

According to Lavigne‐Cruège and Dubourdieu (2002), adding 10 mg/L GSH to the wine during the rapid oxidation test can maintain the color stability and stop the smells from eroding. Additionally, it was also observed that the addition of N‐acetyl cysteine and 20 mg/L GSH prevented the loss of significant volatile compounds (Papadopoulou and Roussis 2008). The decline in the amount of linalool and alpha‐terpineol in muscat wines was inhibited with the addition of glutathione and n‐acetyl cysteine (Kritzinger et al. 2013).

The effect of GSH on wine depends on the grape variety, fermentation, and maturation. Although the tasters in this study did not assert an adverse impact, there have been studies that have assessed both the positive and negative impacts of GSH on wine. Low amounts of glutathione have been shown to preserve flavor components, while high levels have been shown to generate sulfide off‐flavors in wine (Wegmann‐Herr et al. 2016).

## 4 Conclusion

This study examined the substitution of SO_2_ with gGC and GSH in white wine. The findings demonstrate that incorporating gGC and GSH into wine has an impact on the wine's overall composition, aroma components, and organoleptic qualities. However, there was no major difference in the proximate composition of wines supplemented with gGC, GSH, and SO_2_. Furthermore, it has been proven that the inclusion of gGC and GSH in wine proved to be efficacious in protecting phenolic compounds. It is worth noting that the addition of gGC slowed down the browning reactions and resulted in a browning degree value equivalent to SO_2_‐added wine. Furthermore, compared to the control and SO_2_ groups, the addition of gGC and GSH increases the amounts of esters and higher alcohols, which improve the aroma of the wine. Consuming wines that are made without the addition of SO_2_ not only reduces the potential negative effects associated with SO_2_ but also provides a source of gGC or GSH for the human body. Substituting gGC for SO₂ in wine production is recommended based on the positive results obtained in this study.

Further studies should be conducted to assess the ideal usable level of gGC in wines and its synergistic effect with SO₂, evaluate the role of gGC in alcoholic fermentation, investigate its different concentrations, and clarify its impact on the chemical and sensory composition of red and white wines.

## Author Contributions

**Mumine Guruk:** conceptualization (equal), data curation (equal), formal analysis (lead), investigation (lead), methodology (equal), software (equal), validation (equal), writing – original draft (lead), writing – review and editing (equal). **Patrick Fickers:** supervision (equal), writing – review and editing (equal). **Bilal Agirman:** data curation (equal), software (equal), validation (equal), writing – review and editing (equal). **Merve Darıcı:** methodology (equal), validation (equal), writing – review and editing (equal). **Huseyin Erten:** conceptualization (equal), funding acquisition (lead), project administration (lead), resources (equal), supervision (equal), writing – review and editing (equal).

## Conflicts of Interest

The authors declare no conflicts of interest.

## Data Availability

The data that support the findings of this study are available upon reasonable request from the corresponding author.
